# Elevated Hemoglobin A_2_ : A Molecular Revisited, and Implications to β‐Thalassemia Screening

**DOI:** 10.1002/jcla.70294

**Published:** 2026-06-25

**Authors:** Kritsada Singha, Anupong Pansuwan, Hataichanok Srivorakun, Attawut Chaibunruang, Supawadee Yamsri, Wanicha Tepakhan, Kanokwan Sanchaisuriya, Goonnapa Fucharoen, Supan Fucharoen

**Affiliations:** ^1^ Centre for Research and Development of Medical Diagnostic Laboratories, Faculty of Associated Medical Sciences Khon Kaen University Khon Kaen Thailand; ^2^ Biomedical Science Research Unit, Faculty of Medicine Mahasarakham University Mueang Maha Sarakham Mahasarakham Thailand

**Keywords:** diagnostic errors, Hb A2 levels, molecular spectrum, β‐Thalassemia

## Abstract

**Background:**

In Thailand, the Hb A_2_ cut‐off value for β‐thalassemia carrier has been changed from 4.0% to 3.6% since 2015. We examined the molecular basis of β‐thalassemia in a large cohort of Thai subjects with this change.

**Methods:**

A total of 36,313 retrospective specimens encountered during January 2016 to June 2025 were reviewed, and 5909 subjects (16.7%) with Hb A_2_ ≥ 3.6% were recruited. Diagnosis of thalassemia was based on Hb and DNA analyses.

**Results:**

Among the 5909 subjects, 5395 (91.3%) had Hb A_2_A, 115 (1.9%) carried Hb variants, and the remaining 399 (6.8%) resulted from misinterpretations. Of 5395 subjects, 5117 (94.8%) carried β‐thalassemia, 189 (3.5%) had normal β‐globin genes, and 89 (1.6%) had incomplete molecular analysis. Fifty different β‐thalassemia genes were identified, including nine hitherto undescribed in Thailand and two novel mutations, β^CD81(CTC > CTA)^ and β^IVSII‐713(G > A)^. Falsely elevated Hb A_2_ was found in 364 of 399 (70.8%) subjects with misinterpretation, 20 (3.9%) represented human errors, and 15 (2.9%) resulted from Hb E blood transfusion. A low proportion of β^0^‐ and a high proportion of β^+^‐thalassemias, a normal β‐globin gene, and diagnostic errors were observed at borderline Hb A_2_ levels.

**Conclusions:**

A change in the Hb A_2_ cut‐off can alter the molecular spectrum of β‐thalassemia in Thailand. The molecular basis of β‐thalassemia was updated to include 50 mutations, both new and known. Misinterpretation at borderline Hb A_2_ should be aware and cost‐effectiveness should be considered at the screening of β‐thalassemia in the region.

## Graphical abstract

In Thailand, the Hb A_2_ cut‐off value for β‐thalassemia carrier has been changed from 4.0% to 3.6% since 2015. We examined the molecular basis of β‐thalassemia in a large cohort of Thai subjects with this change. The molecular basis of β‐thalassemia was updated, and a change in the Hb A_2_ cut‐off can alter this spectrum.

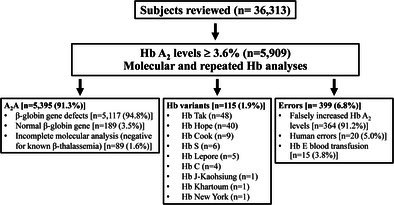

## 1 Introduction

A high prevalence of α‐thalassemia, β‐thalassemia, hemoglobin (Hb) E, and other Hb variants has been reported in Thailand. Interactions between these globin gene defects result in complex genotypes and various thalassemia syndromes, ranging from mild clinical symptoms to severe hemolytic anemia, requiring regular blood transfusion. In Thailand, the prevention and control program for severe thalassemia diseases has been established [1, 2]. In a resource‐limited setting like Thailand, a step‐by‐step approach is necessary to screen target carriers, including α^0^‐thalassemia, β‐thalassemia, and Hb E in at‐risk couples with severe thalassemia diseases. This begins with screening tests using the combined osmotic fragility (OF) test or mean corpuscular volume (MCV), mean corpuscular hemoglobin (MCH), and the Dichlorophenol Indophenol Precipitation (DCIP) test for Hb E. A positive screening test will be followed by Hb and DNA analyses [1, 2, 3].

Β‐thalassemia is an inherited Hb disorder caused by decreased (β^+^‐thalassemia) or absent (β^0^‐thalassemia) β‐globin chain synthesis. Reduced MCV and MCH with elevation of Hb A_2_, characteristics of β‐thalassemia, have been widely utilized for screening of β‐thalassemia [1, 4, 5, 6]. In Thailand, the Hb A_2_ cut‐off has been changed from 4.0% to 3.6% since 2015. It was found, however, that only 5%–10% of these subjects with borderline Hb A_2_ between 3.6%–3.9% were actually β‐thalassemia carriers upon Hb and DNA analyses [7, 8]. While this change of cut‐off value for Hb A_2_ could prevent false negative screening, it could lead to additional workload of Hb and DNA confirmation in a routine setting. In this study, we have addressed this and described an updated molecular spectrum of β‐thalassemia [9, 10, 11, 12], errors arising from misinterpretations, and further implications to thalassemia screening in a large cohort of northeast Thai subjects screened using the changed cut‐off.

## 2 Materials and Methods

### 2.1 Subjects and Hematological Analysis

Ethical approval for this research was obtained from the Institutional Review Board (IRB) of Khon Kaen University, Thailand (HE682074). Informed consent was waived because this is a study of existing data or biological specimens without further prospective data collection from or direct interactions with the patients. From January 2016 to June 2025, a total of 36,313 adult blood specimens were referred to our center for investigation of thalassemia and hemoglobinopathies. A total of 5909 subjects with increased Hb A_2_ levels (Hb A_2_ ≥ 3.6%) were selectively recruited. Hb E‐related disorders were excluded as they have been known to be associated with elevated Hb A_2_ levels and are prevalent in the region [1, 2]. Hematological parameters were measured by standard blood cell counters [Sysmex XN‐9000 system (Sysmex, Kobe, Japan) or DxH 900 Hematology Analyzer (Beckman Coulter Inc., Brea, California, USA)]. Hb analysis was carried out either by using high‐performance liquid chromatography (HPLC) (Variant, Bio‐Rad Laboratory, Hercules, CA, USA) or capillary electrophoresis (Capillarys II Flex Piercing, Sebia, France) at the first visit.

### 2.2 DNA Analysis

Known mutations causing α‐thalassemia, β‐thalassemia, and Hb variants were routinely examined using allele‐specific PCR and GAP‐PCR assays as described [1, 13]. Whole β‐globin sequencing was performed to characterize unknown β‐thalassemia and Hb variants using an ABI PRISM 3730 XL analyzer (Applied Biosystems, Foster City, California, USA) and Barcode‐tagged sequencing (BTSeq, Celemics, Korea). An unknown deletion of the β‐globin gene cluster was screened using the multiplex ligation‐dependent probe amplification (MLPA) assay (SALSA MLPA Probemix P102‐D1 HBB, MRC‐Holland, Amsterdam, the Netherlands), according to the manufacturer's protocol [14]. The β^0^‐thalassemia (Taiwanese deletion) breakpoints were confirmed by GAP‐PCR using primers G9 (5′‐TCCCCAGTTAACCTCCTATT‐3′) and H5 (5′‐GCAGCCTCACCTTCTTTCATGG‐3′), followed by DNA sequencing. KLF1 mutations were identified by PCR‐based methods and DNA sequencing [7].

### 2.3 Statistical Analysis

Data was analyzed using Stata version 18 (STATA, Stata Corp, Texas, USA). The differences between the two independent groups with non‐normal distributions across many variables were compared using the Mann–Whitney U test. Statistical significance was allowed at a *p*‐value < 0.05.

### 2.4 In Silico Prediction Tools

Pathogenicity of the five unstable Hbs and two novel β‐globin mutations found in this study, including Hb Dhonburi, Hb La Desirade, Hb Hezhou, Hb Burke, Hb Crete, β^CD81(CTC > CTA)^ (HBB:c.246C > A), and β^IVSII‐713(G > A)^ (HBB:c.315 + 713G > A), was evaluated using various *in silico* tools as follows: SpliceAI, AlphaMissense, CADD, PhyloP, PolyPhen, PrimateAI‐3D, PromoterAI, REVEL, and SIFT (https://spliceailookup.broadinstitute.org/) [15, 16, 17]. Human splice prediction of the β^CD81(CTC > CTA)^ and β^IVSII‐713(G > A)^, as compared to the wild‐type sequence, was determined on the FGENESH 2.6 program (http://www.softberry.com/berry.phtml) [18] and Neural Network in NNSPLICE 0.9 version (https://www.fruitfly.org/seq_tools/splice.html) [19].

### 2.5 Classification of Diagnostic Errors

Diagnostic errors were defined when subjects with Hb type A_2_A and Hb A_2_ ≥ 3.6% were interpreted as β‐thalassemia carriers at the first visit, but alternatively had a negative result for molecular analysis of β‐thalassemia mutations by DNA analysis. Subjects with Hb A_2_ < 3.6% after the repeated Hb analysis using capillary electrophoresis were defined as falsely elevated Hb A_2_. Since Hb E was initially excluded, those with changes in Hb analysis results to Hb E‐related disorders were defined as human errors. Preanalytical, analytical, and postanalytical errors,e.g., sample mix‐up and data entry errors within a couple, were also classified as human errors [20]. Lastly, when repeated Hb analysis revealed a small peak of Hb E with a negative result for the Hb E gene on DNA analysis, it was suggested that the patient had a history of blood transfusion from Hb E‐carrying donors. Hb E and Hb A_2_ were co‐eluted on HPLC, but they were clearly separated on capillary electrophoresis [21]. The misinterpretation of β‐thalassemia trait, especially on HPLC, due to Hb E blood transfusion, could be detected by capillary electrophoresis.

## 3 Results

**FIGURE 1:**
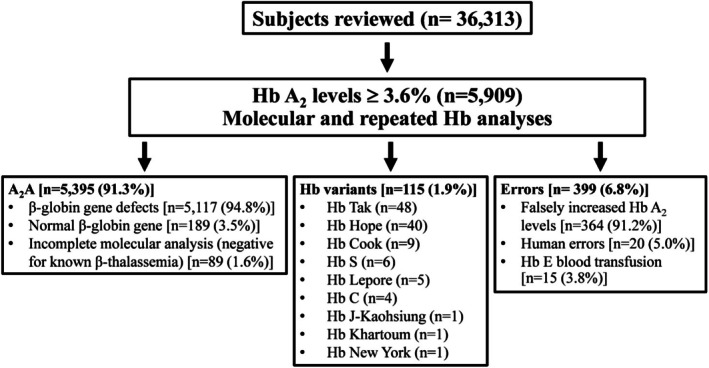
Schematic diagram illustrating the whole subjects reviewed in this study (*n* = 36,313) and molecular characteristics of 5909 subjects with elevated Hb A_2_ levels. These included subjects with Hb A_2_A with Hb A_2_ ≥ 3.6% [*n* = 5395 (91.3%); β‐thalassemia, normal β‐globin gene, unstable Hbs, and incomplete molecular analysis], Hb variants (*n* = 115; 1.9%), and errors [*n* = 399 (6.8%); falsely increased Hb A_2_ levels, human errors, and Hb E blood transfusion].

Of the 36,313 subjects reviewed, 5909 (16.7%) with Hb A_2_ ≥ 3.6% were selected for further analysis. Hb and DNA analyses were performed. For subjects with negative results of molecular analysis, Hb analysis was repeated using capillary electrophoresis. Subjects with repeated Hb A_2_ < 3.6% were interpreted as falsely elevated Hb A_2_, whereas those with elevated Hb A_2_ levels were further investigated by DNA sequencing and followed by MPLA. Figure 1 summarizes the results of this comprehensive investigation. Among the 5909 subjects, 5395 subjects (91.3%) had Hb A_2_A with Hb A_2_ ≥ 3.6%, 115 subjects (1.9%) carried Hb variants, and 399 subjects (6.8%) were those with falsely elevated Hb A_2_. Among those 5395 subjects with Hb A_2_ ≥ 3.6%, most of them [5117 (94.8%)] carried as expected β‐globin gene defects, including non‐deletional β^0^‐thalassemia [*n* = 3613 (66.9%)], large deletional β^0^‐thalassemia [*n* = 211 (39%)], and β^+^‐thalassemia and unstable Hbs [*n* = 1293 (24.0%)]. A total of 189 (3.5%) subjects in this group had an alternatively normal β‐globin gene (Table 1). Unfortunately, due to poor DNA quality, the molecular defects in the remaining 89 subjects (1.6%) could not be completely examined. These subjects tested negative in screening for known β‐thalassemia mutations [1], but further DNA sequencing could not be performed. Several abnormal Hbs were identified in those of 115 subjects with Hb variants. These included Hb Tak (HBB:c.440_441dupAC) (*n* = 48), Hb Hope (HBB:c.410G > A) (*n* = 40), Hb Cook (HBB:c.398A > C) (*n* = 9), Hb S (HBB:c.20A > T) (*n* = 6), Hb Lepore (δβ hybrid) (*n* = 5), Hb C (HBB:c.19G > A) (*n* = 4), Hb J‐Kaohsiung (HBB:c.179A > C) (*n* = 1), Hb Khartoum (HBB:c.374C > G) (*n* = 1), and Hb New York (*n* = 1). For the 399 subjects with investigating errors, we could confirm that 364 (91.2%) had falsely elevated Hb A_2_, 20 (5.0%) were associated with human errors, and 15 (3.8%) were the result of blood transfusion of Hb E carrying blood donors.

**TABLE 1:** Molecular spectrum of 5395 subjects with elevated Hb A_2_ levels, including β‐thalassemia, unstable Hbs, normal β‐globin gene, and incomplete molecular analysis.

β‐globin gene defects	HGVS name (HBB)	Type	*n*	%
**Non‐deletional β^0^‐thalassemia**			**3613**	**66.88**
CD 41/42 (‐CTTT)	c.126_129delCTTT	β^0^	1495	27.71
CD 17 (AAG > TAG)	c.52A > T	β^0^	1014	18.80
IVS I‐1 (G > T)	c.92 + 1G > T	β^0^	342	6.34
IVS I‐5 (G > C)	c.92 + 5G > C	β^0^	245	4.54
CD 71/72 (+A)	c.217dupA	β^0^	166	3.08
IVS II‐654 (C > T)	c.316‐197C > T	β^+^ (severe)	130	2.41
CD 35 (TAC > TAA)	c.108C > A	β^0^	60	1.11
CD 41 (‐C)	c.126delC	β^0^	33	0.61
CD 27/28 (+C)	c.85dupC	β^0^	31	0.57
CD 43 (GAG > TAG)	c.130G > T	β^0^	15	0.28
CD 95 (+A)	c.287dupA	β^0^	12	0.22
105 bp deletion	c.‐74_31del	β^0^	10	0.19
CD 123–125 (−8 bp) (Hb Khon Kaen)	c.370_378delACCCCACCA	β^0^	10	0.19
CD 26 (GAG > TAG)	c.79G > T	β^0^	9	0.17
CD 15 (−T)	c.46delT	β^0^	8	0.15
IVS I‐116 (T > G)	c.93‐15 T > G	β^0^	6	0.11
CD 8/9 (+G)	c.27dupG	β^0^	5	0.09
Init CD (ATG > AGG)	c.2 T > G	β^0^	4	0.07
IVS I‐1 (G > A)	c.92 + 1G > A	β^0^	4	0.07
CD 30 (AGG > ACG)	c.92G > C	β^0^	4	0.07
CD 15 (TGG > TGA)	c.48G > A	β^0^	3	0.06
CD 33/34 (−G)	c.102_103delG	β^0^	2	0.04
IVS I‐2 (T > C)	c.92 + 2 T > C	β^0^	1	0.02
CD 121 (GAA > TAA)^a^	c.364G > T	β^0^	1	0.02
CD 121 (−G) (Hb Mahasarakham)	c.364delG	β^0^	1	0.02
CD 37 (TGG > TAG)^a^	c.113G > A	β^0^	1	0.02
CD 54–58 (−13 bp)^a^	c.165_177delTATGGGCAACCCT	β^0^	1	0.02
**Large‐deletional β^0^‐thalassemia**			**211**	**3.91**
3.4 kb deletion	NG_000007.3:g.69825_73314del	β^0^	191	3.54
118 kb deletion (Filipino deletion)	NC_000011.10:g.5112882_5231358del	β^0^	10	0.19
SEA‐HPFH	NC_000011.10:g.5201647_5229059del	β^0^, HPFH	6	0.11
60 kb deletion (Prachinburi deletion)	NC_000011.10:g.5167971_5,228,123	β^0^	2	0.04
1357 bp deletion (Taiwanese deletion)	NG_000007.3:g.69997_71353del	β^0^	2	0.04
**β^+^‐thalassemia and unstable Hbs**			**1293**	**23.97**
−28 (A > G)	c.‐78A > G	β^+^	773	14.33
CD 19 (AAC > AGC) (Hb Malay)	c.59A > G	β^+^	251	4.65
CD 126 (GTG > GGG) (Hb Dhonburi)	c.380 T > G	β^+^, unstable Hb	105	1.95
−31 (A > G)	c.‐81A > G	β^+^	96	1.78
−87 (C > A)	c.‐137C > A	β^++^	26	0.48
−30 (T > C)	c.‐80 T > C	β^+^	11	0.20
−50 (G > A)	c.‐100G > A	β^++^	9	0.17
−86 (C > G)	c.‐136C > G	β^+^	6	0.11
−90 (C > T)	c.‐140C > T	β^+^	5	0.09
Poly A (A > G) (AATAAA > AATAGA)	c.*112A > G	β^++^	2	0.04
CD 129 (GCC > GTC) (Hb La Desirade)	c.389C > T	Unstable Hb	2	0.04
3′UTR +129 (T > A)^a^	c.*129 T > A	β^++^	1	0.02
−103 (C > A)^a^	c.‐153C > A	β^+^	1	0.02
−88 (C > T)^a^	c.‐138C > T	β^++^	1	0.02
CD18 (GTG > GTA)	c.57G > A	β^+^	1	0.02
CD 64 (GGC > AGC) (Hb Hezhou)^a^	c.193G > A	Unstable Hb	1	0.02
CD 107 (GGC > CGC) (Hb Burke)^a^	c.322G > C	Unstable Hb	1	0.02
CD 129 (GCC > CCC) (Hb Crete)^a^	c.388G > C	Unstable Hb	1	0.02
**Total β‐globin gene defects**			**5117**	**94.85**
**Normal β‐globin gene**			**189**	**3.50**
**Incomplete molecular analysis**			**89**	**1.65**
**Total**			**5395**	**100**

*Note:* Bolded numbers and text represent the total for each subgroup.

^a^ First reported in Thailand.

Table 1 shows an updated molecular basis of β‐thalassemia and related disorders found in this study cohort of northeast Thai patients. Unexpectedly, as many as 50 different β‐globin gene defects were identified among 5117 subjects with elevated Hb A_2_. These included 3825 (74.8%) subjects with β^0^‐thalassemia and 1293 (25.2%) subjects with β^+^‐thalassemia and/or unstable Hbs. Twelve most common β‐thalassemia genes were β^CD41/42(‐CTTT)^, β^CD17(AAG > TAG)^, β^‐^^28(A > G)^, β^IVS I‐1(G > T)^, β^Malay^, β^IVSI‐5(G > C)^, β^3.4 kb deletion^, β^CD71/72(+A)^, β^IVSII‐654(C > T)^, β^Dhonburi^, β^‐^^31(A > G)^, and β^CD35(TAC > TAA)^, covering 95.1% of the mutations found in this area (Table 1). Interestingly, six β‐thalassemia genes [β^3′UTR + 129(T > A)^, β^‐^^103(C > A)^, β^CD121(GAA > TAA)^, β^CD37(TGG > TAG)^, β^‐^^88(C > T)^, and β^CD54‐58(−13bp)^] and three unstable Hbs [Hb Hezhou, Hb Burke, and Hb Crete] were hitherto undescribed in Thailand (See Table 1 footnote a). In addition, two novel mutations, β^CD81(CTC > CTA)^ (HBB:c.246C > A) and β^IVSII‐713(G > A)^ (HBB:c.315 + 713G > A) were identified. All functional prediction programs indicated that these two novel variants are benign (Table S1). Using combined MLPA, GAP‐PCR, and DNA sequencing, a couple with high Hb A_2_ & Hb F phenotype were found to be heterozygotes for the Taiwanese β^0^‐thalassemia deletion (1357 bp deletion). Therefore, they carried a risk of having a fetus with homozygous Taiwanese β^0^‐thalassemia, previously undescribed in Thailand (Figure S1). Genetic counselling was given, and prenatal diagnosis was suggested.

**FIGURE 2:**
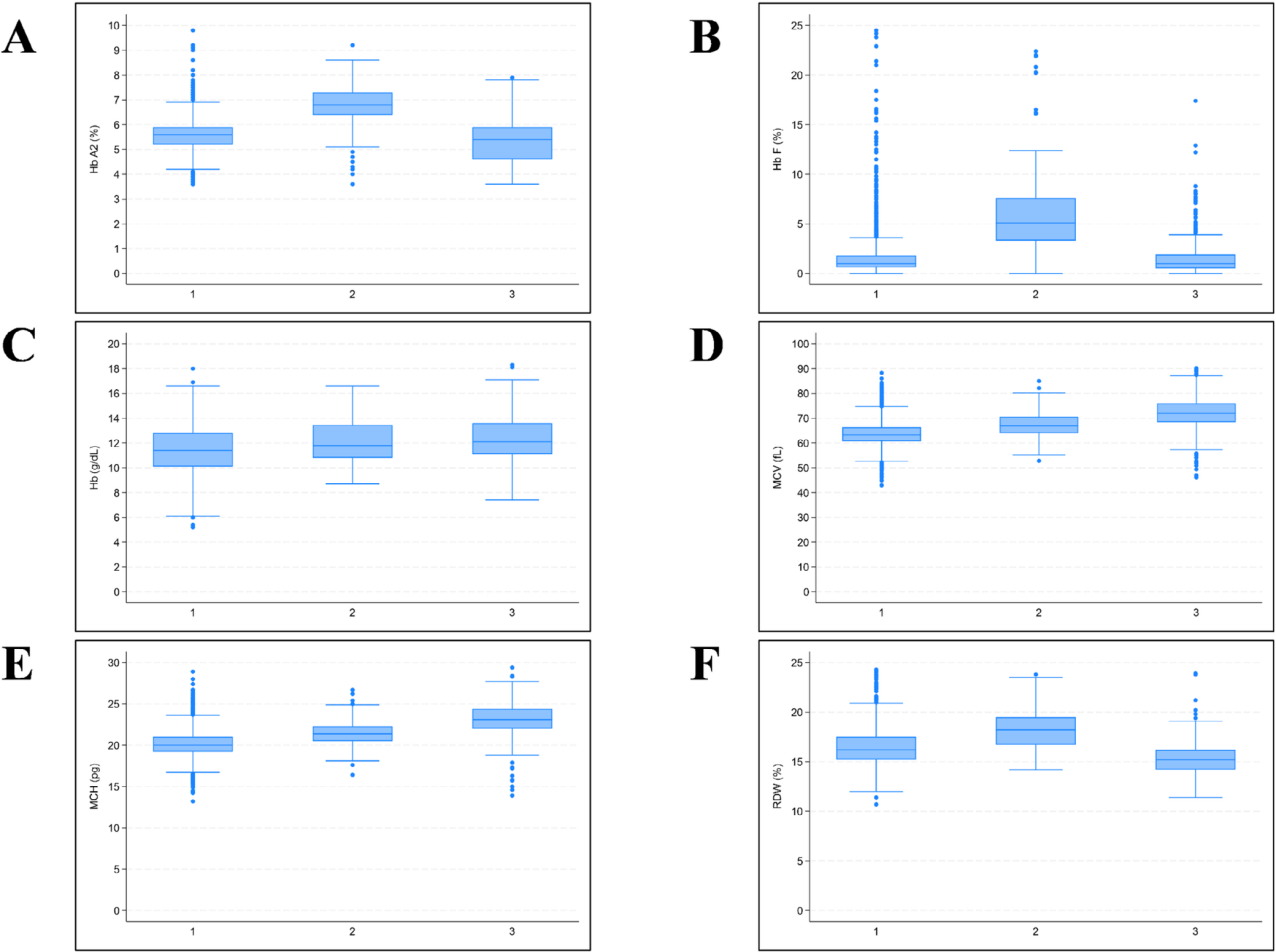
Box plots of Hb A_2_ (A), Hb F (B), Hb (C), MCV (D), MCH (E), and RDW (F) among non‐deletional β^0^‐thalassemia (1), large‐deletional β^0^‐thalassemia (2), and β^+^‐thalassemia and unstable Hbs (3). The solid line across the boxes indicates the median of each group.

Table 2 and Figure 2 showed the comparison of Hb A_2_ & Hb F levels and hematological parameters among non‐deletional β^0^‐thalassemia (*n* = 3613), large deletional β^0^‐thalassemia (*n* = 211), and β^+^‐thalassemia and unstable Hbs (*n* = 1293). Non‐deletional β^0^‐thalassemia had the lowest Hb, Hct, MCV, and MCH, whereas large‐deletional β^0^‐thalassemia had higher Hb A_2_, Hb F, and RDW than other forms. We found that large‐deletional β^0^‐thalassemia was associated with higher MCV and MCH as compared to non‐deletional β^0^‐thalassemia, but had lower MCV and MCH values than those with β^+^‐thalassemia and unstable Hbs. In contrast, those of β^+^‐thalassemia and unstable Hbs had lower Hb A_2_ and higher MCV and MCH as compared to those of non‐deletional and large‐deletional β^0^‐thalassemia groups (See Table 2 footnotes a, a,b, and b).

**TABLE 2:** Comparison of Hb A_2_ and Hb F levels and hematological parameters among non‐deletional β^0^‐thalassemia, large deletional β^0^‐thalassemia, and β^+^‐thalassemia and unstable Hbs.

Groups	*n*	Hb A_2_ (%)	Hb F (%)	RBC (10^12^/L)	Hb (g/dL)	Hct (%)	MCV (fL)	MCH (pg)	MCHC (g/dL)	RDW (%)
Non‐deletional β^0^‐thalassemia	3613	5.6 ± 0.6^a^	1.7 ± 2.3^a^	5.7 ± 0.9^a^	11.5 ± 1.7^a^	36.1 ± 5.6^a^	63.7 ± 4.9^a^	20.2 ± 1.6^a^	31.7 ± 1.4^a^	16.4 ± 2.0^a^
Large‐deletional β^0^‐thalassemia	211	6.7 ± 0.8^a^, ^b^	6.0 ± 4.3^a^, ^b^	5.6 ± 0.8^b^	12.1 ± 1.6^a^	37.7 ± 5.3^a^	67.4 ± 5.0^a^, ^b^	21.5 ± 1.6^a^, ^b^	31.9 ± 1.4^b^	18.2 ± 2.2^a^, ^b^
β^+^‐thalassemia and unstable Hbs	1293	5.2 ± 0.8^a^, ^b^	1.5 ± 1.7^b^	5.3 ± 0.8^a^, ^b^	12.3 ± 1.7^a^	38.3 ± 5.4^a^	72.1 ± 5.5^a^, ^b^	23.2 ± 1.9^a^, ^b^	32.1 ± 1.3^a^, ^b^	15.3 ± 1.6^a^, ^b^

Abbreviations: Hb, hemoglobin; Hct, hematocrit; MCH, mean corpuscular hemoglobin; MCHC, mean corpuscular hemoglobin concentration; MCV, mean corpuscular volume; RBC, red blood cell; RDW, red cell distribution width.

^a^ Significant different from non‐deletional β^0^‐thalassemia in point mutations (*p* < 0.05; Mann–Whitney U‐test).

^b^ Significant different from large deletional β^0^‐thalassemia (*P* < 0.05; Mann–Whitney U‐test).

**FIGURE 3:**
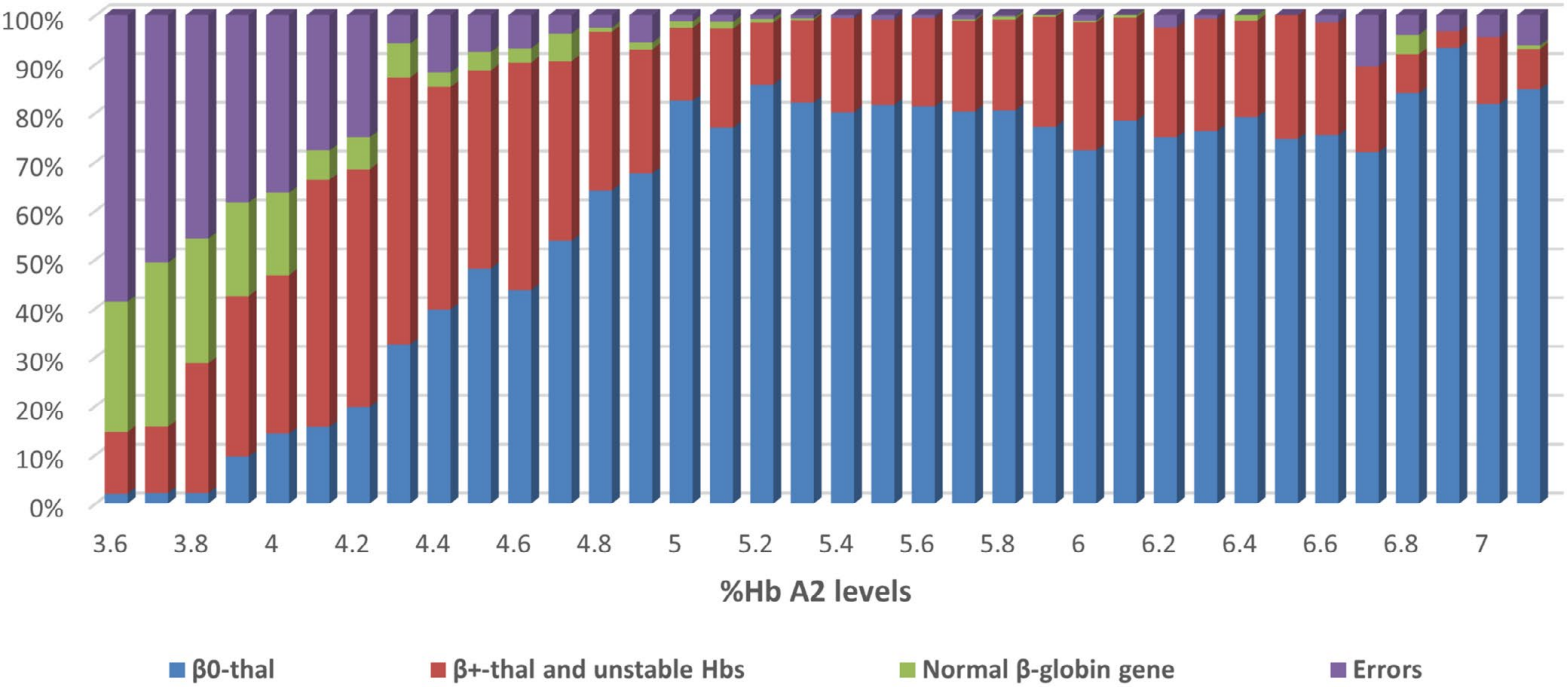
Proportion of β^0^‐thalassemia, β^+^‐thalassemia, normal β‐globin gene, and errors for each Hb A_2_ level. A low proportion of β^0^‐thalassemia and a high proportion of β^+^‐thalassemia, normal β‐globin gene, and errors were found at low Hb A_2_ levels. An increased proportion of β^0^‐thalassemia, a constant proportion of β^+^‐thalassemia, and a decreased proportion of normal β‐globin gene and errors were recognized when Hb A_2_ levels were increased progressively.

Figure 3 and Table S2 demonstrated the proportion of β^0^‐thalassemia, β^+^‐thalassemia, normal β‐globin gene, and errors at each Hb A_2_ level. As shown relatively, a lower proportion of β^0^‐thalassemia and a higher proportion of β^+^‐thalassemia or normal β‐globin gene and errors were observed at lower Hb A_2_ levels. An increased proportion of β^0^‐thalassemia, β^+^‐thalassemia, and a decreased proportion of normal β‐globin gene and errors were observed as Hb A_2_ levels increased progressively. The proportions of β^0^‐thalassemia, β^+^‐thalassemia, or unstable Hbs, normal β‐globin gene, and errors between subjects with Hb A_2_ ≥ 4.0% and Hb A_2_ of 3.6%–3.9% were compared in Table 3. A very high proportion of β^0^‐thalassemia (72.9%) and β^+^‐thalassemia or unstable Hbs (22.8%), and a low proportion of normal β‐globin gene (1.2%) and errors (3.1%) were observed in subjects with Hb A_2_ ≥ 4.0%. In contrast, a very low proportion of β^0^‐thalassemia (2.9%) and a higher proportion of β^+^‐thalassemia or unstable Hbs (16.1%), normal β‐globin gene (28.7%), and errors (52.4%) were observed at the borderline Hb A_2_ levels of 3.6%–3.9%.

**TABLE 3:** Proportion of β^0^‐thalassemia, β^+^‐thalassemia, unstable Hbs, normal β‐globin gene, and errors (falsely raised Hb A_2_ levels, human errors, and Hb E blood transfusion) in each Hb A_2_ group.

Groups	*N* (%)	β^0^‐thal [*n* (%)]	β^+^‐thal/unstable Hbs [*n* (%)]	Normal β‐globin gene [*n* (%)]	Errors [*n* (%)]
Hb A_2_ ≥ 4.0%	5140 (100)	3745 (72.9)	1171 (22.8)	64 (1.2)	160 (3.1)
Hb A_2_ of 3.6%–3.9%	380 (100)	11 (2.9)	61 (16.1)	109 (28.7)	119 (52.4)
MCV < 80 fL	279 (73.4)	10 (3.6)	55 (19.7)	81 (29.0)	133 (47.7)
MCV ≥ 80 fL	101 (26.6)	1 (1.0)	6 (5.9)	28 (27.7)	66 (65.3)
MCH < 27 pg	328 (86.3)	11 (3.4)	59 (18.0)	93 (28.4)	165 (50.3)
MCH ≥ 27 pg	52 (13.7)	0 (0)	2 (3.8)	16 (30.8)	34 (65.4)
MCV < 80 fL or MCH < 27 pg	341 (89.7)	11 (3.2)	60 (17.6)	101 (29.6)	169 (49.6)
MCV ≥ 80 fL and MCH ≥ 27 pg	39 (10.3)	0 (0)	1 (2.6)	8 (20.5)	30 (76.9)

**FIGURE 4:**
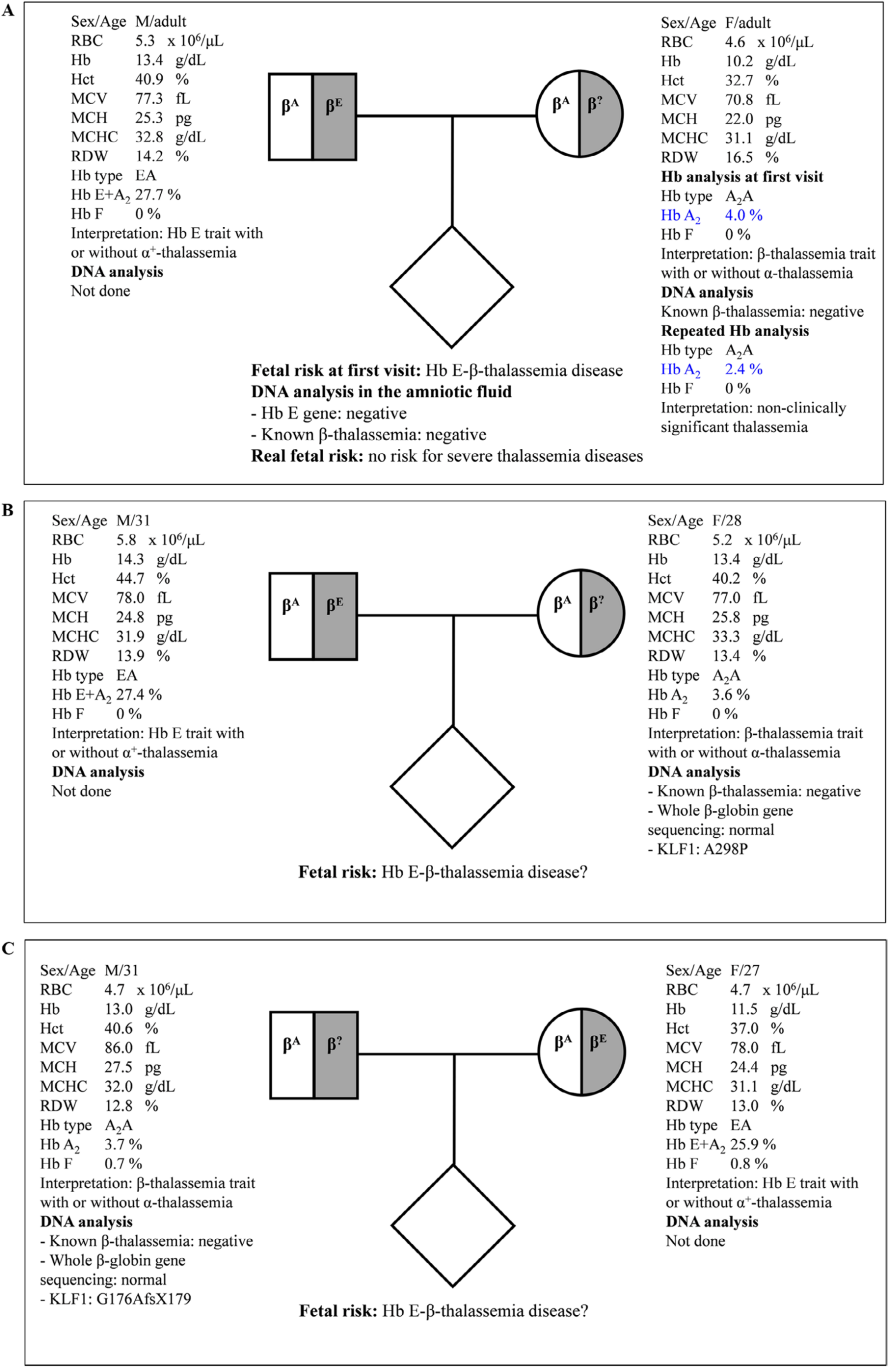
Representative pedigree analysis in couples at risk for Hb E‐β‐thalassemia disease with falsely elevated Hb A_2_ levels (A) and borderline Hb A_2_ levels (B and C). (A) Hematological characteristics indicated that the father had the Hb E trait, and the mother had the β‐thalassemia trait (Hb A_2_ of 4.0%). They were at risk of having a fetus with Hb E‐β‐thalassemia disease. However, no known β‐thalassemia mutations were identified in the mother. Repeat Hb analysis of the mother identified Hb A_2_A, with a normal Hb A_2_ level (2.4%). They had, in fact, no risk of having fetal thalassemia. DNA analysis of amniotic fluid detected no β‐thalassemia and Hb E genes, indicating a normal fetus. This is an example family with unnecessary prenatal diagnosis due to falsely increased Hb A_2_ levels. (B) The father had the Hb E trait, and the mother had a borderline Hb A_2_ level (3.6%). The mother was diagnosed as a β‐thalassemia carrier based on the Hb A_2_ cut‐off of 3.6%, but DNA analysis identified no β‐globin gene mutation. Further investigation of the whole *KLF1* gene in the mother by DNA sequencing identified a heterozygous A298P mutation associated with elevated Hb A_2_ levels. In this family, a couple had no risk for fetal thalassemia disease. (C) The father had borderline Hb A_2_ level (3.7%) with normal MCV (86.0 fL) and MCH (27.5 pg), and the mother was a classical Hb E trait. As for family B, the father was initially diagnosed as a β‐thalassemia trait. Further DNA analysis of the *KLF1* gene in the father identified a G176AfsX179 mutation, associated with elevated Hb A_2_ levels. The fetus had therefore no risk of Hb E‐β‐thalassemia disease. Prenatal diagnosis was not necessary, accordingly.

Interestingly, lower proportions of β^0^‐thalassemia (1.0% and 0% vs. 3.6% and 3.4%) and β^+^‐thalassemia (5.9% and 3.9% vs. 19.7% and 18.0%) were identified in those of subjects with normal MCV and MCH values. We observed a false‐negative result of β^0^‐thalassemia in one subject among 11 subjects (9.1%) with a normal MCV value, although this subject had reduced MCH. This subject with borderline Hb A_2_ (3.9%) and normal MCV (81.5 fL), was found to be double heterozygous β^0^‐thalassemia (β^CD41/42(‐CTTT)^) and α^+^‐thalassemia (3.7 kb deletion). Coinheritance of β‐thalassemia and α‐thalassemia is known to be associated with decreased or normalized Hb A_2_ levels and normalized microcytosis [22]. It is conceivable, therefore, that using decreased MCV or MCH values in individuals with Hb A_2_ 3.6%–3.9% is a better option for screening of β^0^‐thalassemia. It is noteworthy, however, that individuals with this borderline Hb A_2_ may be associated with other genetic variants, e.g., the KLF1 gene [7]. The impact of falsely increased Hb A_2_ and borderline Hb A_2_ levels is shown representatively in Figure 4. In Figure 4A, the father had the Hb E trait, and the mother had the β‐thalassemia trait, a couple at risk of having a fetus with Hb E‐β‐thalassemia disease. However, no known β‐thalassemia mutations were identified in the mother. This prompted us to repeat the Hb analysis of the mother. The repeated analysis identified Hb A_2_A with a normal Hb A_2_ level (2.4% instead of 4.0% initially detected) in the mother and no fetal risk of having Hb E‐β‐thalassemia disease. Amniotic fluid analysis revealed no known β‐thalassemia and Hb E genes. Unnecessary prenatal diagnosis due to falsely increased Hb A_2_ levels was exemplified in this couple. In Figure 4B, the father had the Hb E trait, and the mother had a borderline Hb A_2_ level (3.6%). Whole β‐globin gene sequencing of the mother revealed no β‐globin mutation. Further investigation of the whole *KLF1* gene by DNA sequencing identified a heterozygote for the A298P mutation (NC_000019.10:c.892G > C), previously shown to be associated with elevated Hb A_2_ level [7]. This is also the case for the family shown in Figure 4C, where the father had borderline Hb A_2_ level (3.7%) without β‐globin gene mutation, and the mother had Hb E trait. Further DNA analysis of the *KLF1* gene identified the G176AfsX179 (NC_000019.10:c.525_526insGCGCCGG) mutation. In both families, the fetuses had in fact no risk of having Hb E‐β‐thalassemia disease, and prenatal diagnosis was not necessary.

## 4 Discussion

Molecular analysis of thalassemia is accurate and useful, especially in areas with high prevalence and heterogeneity of thalassemia syndromes like Southeast Asia. However, the associated molecular workflow is usually complex, expensive, and has a long turnaround time, requiring specialized expertise and instruments, which is not suitable for routine practice in the region. Conventional screening using red blood cells and Hb analyses prior to molecular characterization is therefore preferable. We have conducted a comprehensive molecular characterization of β‐thalassemia in a large cohort of Thai subjects with elevated Hb A_2_ (≥ 3.6%) (*n* = 5909) using several techniques, including multiplex allele‐specific PCR and GAP‐PCR assays for the detection of known β‐thalassemia genes [1], and whole β‐globin gene sequencing and MLPA for unknown mutations. Although most of them were β‐thalassemia carriers [5117 of 5909 (86.5%)], some carried alternatively unstable Hbs [*n* = 5 (0.1%)]. In addition, many subjects had a normal β‐globin gene [*n* = 189 (3.2%)]. Mutations in other related genes, such as KLF1, GTF2E2, GATA1, ASH1L, and SUPT5H, and other non‐genetic factors, such as hyperthyroidism, megaloblastic anemia, antiretroviral therapy, pseudoxanthoma elasticum, and hypertrophic osteoarthropathy, may play roles with increased Hb A_2_ in this group of subjects with normal β‐globin gene [4, 5, 6, 7, 23, 24]. Further investigation of these genetic and non‐genetic factors in this group of subjects should provide additional information related to the elevation of Hb A_2_ in non‐β‐thalassemia carriers.

Using a cut‐off of Hb A_2_ ≥ 4.0% for detecting β‐thalassemia carriers, the molecular basis of β‐thalassemia in northeast Thailand was reported in 2011 [9]. Seventeen β‐thalassemia genes were identified. The β^CD41/42(‐CTTT)^, β^CD17(AAG > TAG)^, β^‐^^28(A > G)^, β^CD71/72(+A)^, β^IVS I‐1(G > T)^, β^IVSII‐654(C > T)^, β^3.4 kb deletion^, β^IVSI‐5(G > C)^, β^CD43(GAG > TAG)^, and β^CD35(TAC > TAA)^, were among the most common mutations, comprising 98.6% of the β‐thalassemia genes found. The frequency of β^0^‐thalassemia was 77.0%, and that of β^+^‐thalassemia was 23.0%. This information has now been updated in our study on a larger cohort of subjects after adjusting the Hb A_2_ cut‐off from 4.0% to 3.6% [7, 8]. As many as 50 different mutations were identified. Increased proportions of β^Malay^, β^Dhonburi^, and β^‐^^31(A > G)^, all of which are β^+^‐thalassemia, were obviously observed (Table 1). In fact, this is not unexpected since these β^+^‐thalassemia genes are associated with lower expression of Hb A_2_ as compared to other β‐thalassemia mutations, with an average Hb A_2_ of 5.5% ± 0.7%. It is noteworthy that other β^+^‐thalassemia mutations, e.g., β^‐^^28(A > G)^, β^‐^^87(C > A)^, β^‐^^86(C > G)^, and β^‐^^90(C > T)^ had higher Hb A_2_ expression (averaging 5.6%–5.8%) than β^Malay^, β^Dhonburi^, β^‐^^31(A > G)^, β^‐^^30(T > C)^, and β^‐^^50(G > A)^ (averaging 4.2%–4.6%). In our series, when using a cut‐off Hb A_2_ of 3.6%, we observed a slight increase in the proportion of β^+^‐thalassemia and unstable Hbs [1293 of 5117 (25.3%)], as compared to those with the cut‐off of 4.0% [1171 in 4916 (23.8%)]. This likely indicates that, unlike β^0^‐thalassemia, carriers of some types of β^+^‐thalassemia and unstable Hbs are associated with a slightly lower elevation of Hb A_2_.

Interestingly, we found two novel β‐thalassemia mutations [the β^CD81(CTC > CTA)^ and the β^IVSII‐713(G > A)^] and nine previously undescribed variants in Thailand. It is noteworthy that a novel mutation at codon 81, CTC (Leu) to CTA (Leu), does not lead to an amino acid substitution. Human splicing site prediction showed no difference between the two novel mutations and the wild‐type sequence, either. Moreover, *in silico* tools predicted that the two novel variants were both associated with benign abnormalities (Table S1). Therefore, these two novel mutations likely represent mild β‐thalassemia alleles.

In our study, five Hb variants were identified, namely, Hb Dhonburi, Hb La Desirade, Hb Burke, Hb Crete, and Hb Hezhou. The first four variants have been described as unstable Hbs [25, 26, 27, 28]. Among them, Hb Dhonburi is the most common one, being identified in 105 of 1293 subjects with β^+^‐thalassemia (Figure 1 & Table 2). Hb Hezhou, found originally in two Chinese heterozygotes, had a normal clinical presentation and normal isopropanol precipitation test [29]. However, a Thai subject with Hb Hezhou in our study had a positive DCIP test, the screening test for Hb E and unstable Hbs [2, 3], the data indicating an instability of the Hb Hezhou molecule. A prediction study revealed that Hb Hezhou is associated with a pathogenic variant in most functional studies (Table S1). Since all these Hb variants were associated with elevated Hb A_2_ levels, they could be misinterpreted as heterozygous β‐thalassemia [30]. Therefore, care should be taken in the interpretation of these Hb variants in a routine setting, and where possible, molecular characterization is recommended.

As shown in Table 2, five large β‐globin gene deletions, including the 3.4 kb deletion, 118 kb deletion (Filipino deletion), SEA‐HPFH deletion, 60 kb Prachinburi deletion, and 1357 bp Taiwanese deletion, were identified in this study cohort. All of them have been described in Thailand [31, 32, 33, 34, 35, 36]. We found that the 3.4 kb deletion is the most common one in this study, being identified in 191 of 205 subjects with deletional β‐thalassemia. These 3.4 kb deletion, 118 kb Filipino deletion, SEA‐HPFH, 60 kb Prachinburi deletion, and 1357 bp Taiwanese deletion are associated with high Hb A_2_ and high Hb F β‐thalassemia trait, although the SEA‐HPFH has been noted to be associated with HPFH rather than β‐thalassemia in some studies [31, 32, 33, 34, 35, 36]. It is also noteworthy that these large gene deletions may pose a diagnostic challenge because they cannot be characterized by DNA sequencing. Moreover, most deletional β^0^‐thalassemia is associated with milder clinical severity in Hb E‐β^0^‐thalassemia and homozygous β‐thalassemia diseases as compared to non‐deletional β^0^‐thalassemia [34, 35]. Accordingly, in some studies, they were classified as β^+^‐thalassemia alleles [11]. Identification of these β‐thalassemia deletions by a specific PCR assay is important to genetic counselling, especially in couples at risk for severe thalassemia diseases.

Several Hb analyzers have been utilized in Thailand, yielding satisfactory results for Hb analysis, including HPLC, capillary zone electrophoresis (CZE), and capillary isoelectric focusing (cIEF) [13, 37, 38]. The precision and accuracy of Hb A_2_ measurement are important for β‐thalassemia diagnosis. A relative total error of 7.0% has been proposed to classify β‐thalassemia and non‐β‐thalassemia accurately. Thus, for Hb A_2_ of 3.6%, the Hb A_2_ quantification should be within 3.6% ± 0.25% (3.35%–3.85%) [4]. Variability of Hb A_2_ quantification among automated Hb analyzers has been noted. The primary analytical factors affecting Hb A_2_ measurement include incorrect buffer or column temperature, inappropriate sample application (too small or too large) across all methods, and improper baseline or integration in HPLC [4, 5, 6]. The errors associated with elevated Hb A_2_ levels were demonstrated at 6.8% (Figure 1). These should lead to misdiagnosis, increase further workload, and unnecessary expense, and probably unnecessary prenatal diagnosis [39]. In this study, we unexpectedly observed a high error rate at borderline Hb A_2_ levels of 3.6%–3.9% [230 of 457 (50.3%)] as compared to that of Hb A_2_ ≥ 4.0% [160 in 5,100140 (3.1%)]. Falsely increased Hb A_2_ levels were the most common errors [*n* = 364 (6.2%)], which could be related to the variable Hb A_2_ quantification among automated Hb analyzers [4, 5, 6]. The impact of falsely elevated Hb A_2_ levels was demonstrated in a family shown in Figure 4A, leading to unnecessary prenatal diagnosis and fetal tissue sampling. Human errors [*n* = 20 (0.3%)] at preanalytical, analytical, and postanalytical processes, like switching of hematological parameters, and samples are among the major human errors. Lastly, blood transfusion from Hb E‐carrying donors could cause false elevation of Hb A_2_ and a misinterpretation of β‐thalassemia trait, especially on HPLC, which could not separate Hb E and Hb A_2_ [*n* = 15 (0.3%)] [21]. Thus, blood transfusion history is necessary for the interpretation of Hb analysis. It is worth mentioning that all errors were typically detected after the result of negative molecular analysis and repeated Hb analysis.

In general, β^+^‐thalassemia causes milder β‐thalassemia disease than that of β^0^‐thalassemia [40]. Therefore, β^0^‐thalassemia is a main target of thalassemia screening in at‐risk couples for severe β‐thalassemia diseases in Thailand, such as homozygous β^0^‐thalassemia (β^0^/β^0^) and Hb E‐β^0^‐thalassemia (β^E^/β^0^) [1, 2]. Although β^+^‐thalassemia is not the target carrier at thalassemia screening, Hb E‐β^+^‐thalassemia (β^E^/β^+^) and compound heterozygous β^0^‐thalassemia and β^+^‐thalassemia (β^0^/β^+^) had significant clinical symptoms, ranging from mild to transfusion‐dependent [41, 42]. Screening for both β^+^‐thalassemia and β^0^‐thalassemia is therefore essential. However, based on our study, the cost‐effectiveness should be taken into consideration at borderline Hb A_2_ levels (3.6%–3.9%). We found a very low proportion of β^0^‐thalassemia (2.9%) and a high proportion of β^+^‐thalassemia (16.1%) or normal β‐globin gene (28.7%), and investigation errors (52.4%) (Table 2). In addition, it was found that more than 80% of subjects with borderline Hb A_2_ levels had no β‐thalassemia mutation. It is conceivable, therefore, that this could lead to further workload of DNA sequencing, expense, difficulty in genetic counselling, and unnecessary prenatal diagnosis in at‐risk couples [39], as exemplified in Figure 4A–C. Fortunately, based on our study, reduced MCH would detect all β^0^‐thalassemia carriers with borderline Hb A_2_, whereas MCV value would, in contrast, yield a small number of false negatives (9.1%). Although MCH is more effective than MCV in thalassemia screening and has lower variability than MCV among blood cell analyzers, it is less popular and is not widely used in Thailand [43]. We recommend using both reduced MCV and reduced MCH as markers for effective screening of β^+^ and β^0^‐thalassemias (Table 3). It is noteworthy that using non‐invasive prenatal testing (NIPT) of cell‐free fetal DNA may be an alternative option to prevent unnecessary prenatal diagnosis for at‐risk couples to rule out an affected fetus with safe and accurate results, before resorting to invasive prenatal testing [44].

In summary, changing the Hb A_2_ cut‐off from 4.0% to 3.6% could modify the molecular spectrum of β‐thalassemia in Thailand. The molecular basis in a large cohort was updated with as many as 50 different mutations, including both new and known mutations. Errors associated with misinterpretations of Hb A_2_, including Hb variants, falsely increased Hb A_2_ levels, human errors, and Hb E blood transfusion, should be carefully addressed in a routine setting. Cost‐effectiveness should also be considered in subjects with borderline Hb A_2_ levels, given the very low prevalence of β^0^‐thalassemia in this range of Hb A_2_ levels. For accurate interpretation of thalassemia in an endemic area with heterogeneity of thalassemia and hemoglobinopathies like Thailand, we confirm that it is necessary to use all laboratory findings, including initial screening, Hb analysis confirmation, and molecular testing. Yet, this notation is limited to Thailand with high prevalence and heterogeneity of thalassemia and may not be directly extrapolated to other populations. The performance characteristics of the Hb A_2_ cut‐off should be highly population‐specific due to the different mutations of thalassemia and hemoglobinopathies and modifier genes [45].

## Author Contributions

Kritsada Singha designed the study, performed experiments, analyzed the data, and developed the initial manuscript. Anupong Pansuwan, Hataichanok Srivorakun, Attawut Chaibunruang, Supawadee Yamsri, and Wanicha Tepakhan assisted with routine genetic analysis of the cases. Goonnapa Fucharoen and Kanokwan Sanchaisuriya analyzed the data and interpretation of the cases. Supan Fucharoen supervised results interpretation, designed and facilitated the study, acquired a research grant, and critically revised and approved the final manuscript. All authors approved the final submitted version.

## Funding

This study was financially supported by the Fundamental Fund of Khon Kaen University, under the National Science, Research and Innovation Fund (NSRF), Thailand, to Supan Fucharoen (Contract ID: FF2569 KKU), and Genomics Thailand, the Health System Research Institute (Contract ID: HSRI 68‐049), and Faculty of Medicine, Mahasarakham University, Thailand, to Kritsada Singha.

## Ethics Statement

Ethical approval for this research was obtained from the Institutional Review Board (IRB) of Khon Kaen University, Thailand (HE682074). Informed consent was waived because this is a study of existing data or biological specimens without further prospective data collection from or direct interactions with the patients.

## Conflicts of Interest

The authors declare no conflicts of interest.

## Supporting information


**Table S1:** The *in silico* functional predictors of the five unstable Hbs and two novel β‐globin variants.
**Table S2:** Numbers and proportions of β0‐thalassemia, β+‐thalassemia, and unstable Hbs, normal β‐globin gene, and errors for each Hb A2 level. Subjects with incomplete molecular analysis, Hb variants, and imprecise Hb A2 levels (range of data) were excluded.
**Figure S1:** A couple with a risk of having a homozygous β0‐thalassemia fetus and confirmation of the Taiwanese β0‐thalassemia by MLPA, GAP‐PCR, and DNA sequencing. (A): Pedigree analysis of a couple with heterozygous Taiwanese β0‐thalassemia. (B): MLPA analysis indicating a heterozygous deletion extending from probe HBB‐1‐148 nt to HBB‐Intr2‐196 nt with the threshold ratio at < 0.7. (C): GAP‐PCR analysis using primers G9 and H5 for identification of the Taiwanese β0‐thalassemia deletion (1219 bp instead of a normal 2,576 bp). M represents the Lambda DNA/HindIII Marker. Lanes 1 and 3: Normal control, and lanes 2 and 4: Heterozygous Taiwanese β0‐thalassemia. (D): DNA sequencing across the deletion breakpoint of the Taiwanese β0‐thalassemia with NG_000007.3:g.69997_71353del.

## Data Availability

The data that support the findings of this study are available on request from the corresponding author. The data are not publicly available due to privacy or ethical restrictions.

## References

[1] S. Yamsri , K. Sanchaisuriya , G. Fucharoen , N. Sae‐Ung , T. Ratanasiri , and S. Fucharoen , “Prevention of Severe Thalassemia in Northeast Thailand: 16 Years of Experience at a Single University Center,” Prenatal Diagnosis 30 (2010): 540–546, 10.1002/pd.2514. 20509153

[2] S. Fucharoen and D. J. Weatherall , “Progress Toward the Control and Management of the Thalassemias,” Hematology/Oncology Clinics of North America 30 (2016): 359–371, 10.1016/j.hoc.2015.12.001. 27040959

[3] G. Fucharoen , K. Sanchaisuriya , N. Sae‐ung , S. Dangwibul , and S. Fucharoen , “A Simplified Screening Strategy for Thalassaemia and Haemoglobin E in Rural Communities in South‐East Asia,” Bulletin of the World Health Organization 82 (2004): 364–372. PMCID: PMC2622836. 15298227 PMC2622836

[4] A. Mosca , R. Paleari , G. Ivaldi , R. Galanello , and P. C. Giordano , “The Role of Haemoglobin A(2) Testing in the Diagnosis of Thalassaemias and Related Haemoglobinopathies,” Journal of Clinical Pathology 62 (2009): 13–17, 10.1136/jcp.2008.056945. 19103851

[5] A. D. Stephens , M. Angastiniotis , E. Baysal , et al., “ICSH Recommendations for the Measurement of Haemoglobin A2,” International Journal of Laboratory Hematology 34 (2012): 1–13, 10.1111/j.1751-553X.2011.01368.x. 21974826

[6] S. Thilakarathne , U. P. Jayaweera , and A. Premawardhena , “Unresolved Laboratory Issues of the Heterozygous State of β‐Thalassemia: A Literature Review,” Haematologica 109 (2024): 23–32, 10.3324/haematol.2022.282667. 37259577 PMC10772521

[7] H. Srivorakun , W. Thawinan , G. Fucharoen , K. Sanchaisuriya , and S. Fucharoen , “Thalassemia and Erythroid Transcription Factor KLF1 Mutations Associated With Borderline Hemoglobin A_2_ in the Thai Population,” Archives of Medical Science 18 (2020): 112–120, 10.5114/aoms.2020.93392. 35154532 PMC8827018

[8] P. Chaweephisal , A. Phusua , K. Fanhchaksai , S. Sirichotiyakul , and P. Charoenkwan , “Borderline Hemoglobin A_2_ Levels in Northern Thai Population: HBB Genotypes and Effects of Coinherited Alpha‐Thalassemia,” Blood Cells, Molecules & Diseases 74 (2019): 13–17, 10.1016/j.bcmd.2018.10.002. 30309760

[9] S. Yamsri , K. Sanchaisuriya , G. Fucharoen , N. Sae‐Ung , and S. Fucharoen , “Genotype and Phenotype Characterizations in a Large Cohort of β‐Thalassemia Heterozygote With Different Forms of α‐Thalassemia in Northeast Thailand,” Blood Cells, Molecules & Diseases 47 (2011): 120–124, 10.1016/j.bcmd.2011.05.003. 21664157

[10] C. Nopparatana , C. Nopparatana , V. Saechan , S. Karnchanaopas , and K. Srewaradachpisal , “Prenatal Diagnosis of α‐ and β‐Thalassemias in Southern Thailand,” International Journal of Hematology 111 (2020): 284–292, 10.1007/s12185-019-02761-4. 31659625

[11] V. Viprakasit , C. Limwongse , S. Sukpanichnant , et al., “Problems in Determining Thalassemia Carrier Status in a Program for Prevention and Control of Severe Thalassemia Syndromes: A Lesson From Thailand,” Clinical Chemistry and Laboratory Medicine 51 (2013): 1605–1614, 10.1515/cclm-2013-0098. 23525874

[12] P. Panichchob , P. Iamdeelert , P. Wongsariya , et al., “Molecular Spectrum of β‐Thalassemia Mutations in Central to Eastern Thailand,” Hemoglobin 45 (2021): 97–102, 10.1080/03630269.2021.1924193. 33966551

[13] H. Srivorakun , K. Singha , G. Fucharoen , K. Sanchaisuriya , and S. Fucharoen , “A Large Cohort of Hemoglobin Variants in Thailand: Molecular Epidemiological Study and Diagnostic Consideration,” PLoS One 9 (2014): e108365, 10.1371/journal.pone.0108365. 25244406 PMC4171515

[14] M. Phylipsen , J. F. Prior , E. Lim , et al., “Thalassemia in Western Australia: 11 Novel Deletions Characterized by Multiplex Ligation‐Dependent Probe Amplification,” Blood Cells, Molecules & Diseases 44 (2010): 146–151, 10.1016/j.bcmd.2009.12.011. 20110179

[15] J. M. de Sainte Agathe , M. Filser , B. Isidor , et al., “SpliceAI‐Visual: A Free Online Tool to Improve SpliceAI Splicing Variant Interpretation,” Human Genomics 17 (2023): 7, 10.1186/s40246-023-00451-1. 36765386 PMC9912651

[16] V. Pejaver , A. B. Byrne , B. J. Feng , et al., “Calibration of Computational Tools for Missense Variant Pathogenicity Classification and ClinGen Recommendations for PP3/BP4 Criteria,” American Journal of Human Genetics 109 (2022): 2163–2177, 10.1016/j.ajhg.2022.10.013. 36413997 PMC9748256

[17] T. Bergquist , S. L. Stenton , E. A. W. Nadeau , et al., “Calibration of Additional Computational Tools Expands ClinGen Recommendation Options for Variant Classification With PP3/BP4 Criteria,” Genetics in Medicine 27 (2025): 101402, 10.1016/j.gim.2025.101402. 40084623 PMC12208618

[18] M. G. Reese , F. H. Eeckman , D. Kulp , and D. Haussler , “Improved Splice Site Detection in Genie,” Journal of Computational Biology 4 (1997): 311–323, 10.1089/cmb.1997.4.311. 9278062

[19] V. Solovyev , P. Kosarev , I. Seledsov , and D. Vorobyev , “Automatic Annotation of Eukaryotic Genes, Pseudogenes and Promoters,” Genome Biology 7, no. Suppl 1 (2006): 1–12, 10.1186/gb-2006-7-s1-s10. PMC1810547 16925832

[20] M. Plebani , “The Detection and Prevention of Errors in Laboratory Medicine,” Annals of Clinical Biochemistry 47 (2010): 101–110, 10.1258/acb.2009.009222. 19952034

[21] N. Sae‐ung , H. Srivorakun , G. Fucharoen , S. Yamsri , K. Sanchaisuriya , and S. Fucharoen , “Phenotypic Expression of Hemoglobins A_2_, E and F in Various Hemoglobin E Related Disorders,” Blood Cells, Molecules & Diseases 48 (2012): 11–16, 10.1016/j.bcmd.2011.09.008. 22014901

[22] N. Saleh‐Gohari , M. Khademi Bami , R. Nikbakht , and H. Karimi‐Maleh , “Effects of α‐Thalassaemia Mutations on the Haematological Parameters of β‐Thalassaemia Carriers,” Journal of Clinical Pathology 68 (2015): 562–566, 10.1136/jclinpath-2014-202825. 25935548

[23] C. L. Harteveld , A. Achour , N. F. Fairuz Mohd Hasan , et al., “Loss‐Of‐Function Variants in SUPT5H as Modifying Factors in Beta‐Thalassemia,” International Journal of Molecular Sciences 25 (2024): 8928, 10.3390/ijms25168928. 39201615 PMC11354595

[24] A. Achour , T. Koopmann , R. Castel , et al., “A New Gene Associated With a β‐Thalassemia Phenotype: The Observation of Variants in SUPT5H,” Blood 136 (2020): 1789–1793, 10.1182/blood.2020005934. 32589702

[25] P. Kountouris , C. W. Lederer , P. Fanis , X. Feleki , J. Old , and M. Kleanthous , “IthaGenes: An Interactive Database for Haemoglobin Variations and Epidemiology,” PLoS One 9 (2014): e103020, 10.1371/journal.pone.0103020. 25058394 PMC4109966

[26] P. Kamseng , S. Trakulsrichai , O. Trachoo , et al., “Low Oxygen Saturation and Severe Anemia in Compound Heterozygous Hb Louisville [β42(CD1)Phe→Leu] and Hb La Desirade [β129(H7)ala→Val],” Hematology 22 (2017): 114–118, 10.1080/10245332.2016.1231989. 27670359

[27] S. Kobayashi , T. Nara , Y. Nakano , et al., “Hemoglobin Burke: An Unstable Hemoglobin Rarely Associated With Hemolytic Episodes,” Hemoglobin 10 (1986): 661–666, 10.3109/03630268609036570. 3557997

[28] I. Papassotiriou , J. Traeger‐Synodinos , M. C. Marden , et al., “The Homozygous State for Hb Crete [beta129 (H7) Ala Pro] Is Associated With a Complex Phenotype Including Erythrocytosis and Functional Anemia,” Blood Cells, Molecules & Diseases 34 (2005): 229–234, 10.1016/j.bcmd.2004.12.006. 15885607

[29] Y. L. Zhao , Q. F. Lin , X. W. He , Y. Q. Li , and L. Liang , “Hb Hezhou [β64(E8)Gly→Ser; HBB: C.193G>A]: A Novel Variant on the β‐Globin Gene,” Hemoglobin 45 (2021): 133–135, 10.1080/03630269.2021.1908347. 33843396

[30] K. Singha , H. Srivorakun , S. Yamsri , et al., “Common Hemoglobin Variants Affecting the Diagnosis of β‐Thalassemia: A Large Cohort Data at a Single Center,” PLoS One 21 (2026): e0346729, 10.1371/journal.pone.0346729. 41996320 PMC13089717

[31] S. Yamsri , K. Sanchaisuriya , G. Fucharoen , and S. Fucharoen , “Genetic Origin and Interaction of the Filipino β^0^‐Thalassemia With Hb E and α‐Thalassemia in a Thai Family,” Translational Research 159 (2012): 473–476, 10.1016/j.trsl.2011.10.008. 22633098

[32] K. Singha , W. Tepakhan , S. Yamsri , et al., “A Large Cohort of Deletional High Hemoglobin F Determinants in Thailand: A Molecular Revisited and Identification of a Novel Mutation,” Clinica Chimica Acta 551 (2023): 117615, 10.1016/j.cca.2023.117615. 37884119

[33] W. Jomoui and W. Tepakhan , “Characterization and Identification of Prachinburi β^0^ ‐Thalassemia: A Novel‐60 Kb Deletion in Beta Globin Gene Related to High Levels of Hb F in Heterozygous State,” International Journal of Laboratory Hematology 43 (2021): O200‐3, 10.1111/ijlh.13511. 33734585

[34] C. Soontornpanawet , K. Singha , H. Srivorakun , W. Tepakhan , G. Fucharoen , and S. Fucharoen , “Molecular Basis of a High Hb A_2_/Hb F β‐Thalassemia Trait: A Retrospective Analysis, Genotype‐Phenotype Interaction, Diagnostic Implication, and Identification of a Novel Interaction With α‐Globin Gene Triplication,” PeerJ 11 (2023): e15308, 10.7717/peerj.15308. 37159832 PMC10163868

[35] C. Soontornpanawet , K. Singha , and S. Fuchareon , “Genotype and Phenotype Characteristics of Homozygous and Compound Heterozygous β‐Thalassemia With 3.4 Kb Deletion,” International Journal of Laboratory Hematology 44 (2022): e230‐2, 10.1111/ijlh.13900. 35644030

[36] K. Traisrisilp , Y. Zheng , K. W. Choy , and P. Chareonkwan , “Thalassemia Screening by Third‐Generation Sequencing: Pilot Study in a Thai Population,” Obstet Med 17 (2024): 101–107, 10.1177/1753495X231207676. 38784187 PMC11110746

[37] H. Srivorakun , G. Fucharoen , K. Sanchaisuriya , and S. Fucharoen , “Diagnosis of Common Hemoglobinopathies Among South East Asian Population Using Capillary Isoelectric Focusing System,” International Journal of Laboratory Hematology 39 (2017): 101–111, 10.1111/ijlh.12585. 27981786

[38] S. Satthakarn , K. Panyasai , A. Phasit , and S. Panyasai , “Reliability of Hemoglobin A_2_ Value as Measured by the Premier Resolution System for Screening of β‐Thalassemia Carriers,” Clinical Chemistry and Laboratory Medicine 62 (2023): 453–463, 10.1515/cclm-2023-1006. 37845805

[39] K. Singha , S. Yamsri , A. Chaibunruang , et al., “Frequency of Unnecessary Prenatal Diagnosis of Hemoglobinopathies: A Large Retrospective Analysis and Implication to Improvement of the Control Program,” PLoS One 18 (2023): e0283051, 10.1371/journal.pone.0283051. 37058522 PMC10104333

[40] S. Yamsri , K. Singha , T. Prajantasen , et al., “A Large Cohort of β(+)‐thalassemia in Thailand: Molecular, Hematological and Diagnostic Considerations,” Blood Cells, Molecules & Diseases 54 (2015): 164–169, 10.1016/j.bcmd.2014.11.008. 25471338

[41] V. Viprakasit and S. Ekwattanakit , “Clinical Classification, Screening and Diagnosis for Thalassemia,” Hematology/Oncology Clinics of North America 32 (2018): 193–211, 10.1016/j.hoc.2017.11.006. 29458726

[42] P. Rukwong , R. Natesirinilkul , L. Sathitsamitphong , et al., “Clinical and Hematological Characteristics of Beta‐Plus Thalassemia and Uncommon Beta‐Chain Hemoglobin Variants in Northern Thailand,” Annals of Medicine 57 (2025): 2551815, 10.1080/07853890.2025.2551815. 40888401 PMC12404072

[43] C. Chaitraiphop , K. Sanchaisuriya , S. Inthavong , et al., “Thalassemia Screening Using Different Automated Blood Cell Counters: Consideration of Appropriate Cutoff Values,” Clinical Laboratory 62 (2016): 545–552, 10.7754/clin.lab.2015.150720. 27215072

[44] L. Xiong , A. N. Barrett , R. Hua , et al., “Non‐Invasive Prenatal Testing for Fetal Inheritance of Maternal β‐Thalassaemia Mutations Using Targeted Sequencing and Relative Mutation Dosage: A Feasibility Study,” BJOG 125 (2018): 461–468, 10.1111/1471-0528.15045. 29211324

[45] D. J. Weatherall , “The Inherited Diseases of Hemoglobin Are an Emerging Global Health Burden,” Blood 115 (2010): 4331–4336, 10.1182/blood-2010-01-251348. 20233970 PMC2881491

