# Supplementary material for: Elevated Hemoglobin A2 : A Molecular Revisited, and Implications to β‐Thalassemia Screening
Source: J Clin Lab Anal. 2026 Jun 25;40(15):e70294. doi: 10.1002/jcla.70294 (PMC13399722; doi:10.1002/jcla.70294)
Supplement: Supplementary file 1 [file JCLA-40-e70294-s001.docx]

**Table S1** The *in silico* functional predictors of the five unstable Hbs and two novel β-globin variants

| **Variants** | **HGVS name**  **(HBB)** | **SpliceAI^a^** | **AlphaMissense^b^** | **CADD^b^** | **PhyloP^b^** | **PolyPhen^b^** | **PrimateAI-3D^c^** | **PromoterAI^d^** | **REVEL^b^** | **SIFT^b^** |
| --- | --- | --- | --- | --- | --- | --- | --- | --- | --- | --- |
| Hb Dhonburi | c.380T>G | 0.04 | 0.2818  (Indeterminate) | 13.8  (Moderate Benign) | -2.04  (Moderate Benign) | 0.226  (Indeterminate) | 0.47  (likely deleterious) | - | 0.67  (Supporting Pathogenic) | 0.01  (Indeterminate) |
| Hb La Desirade | c.389C>T | 0.01 | 0.3754  (Indeterminate) | 22.6  (Supporting Benign) | 1.74  (Supporting Benign) | 0.025  (Supporting Benign) | 0.32  (Benign) | - | 0.818  (Moderate Pathogenic) | 0.04  (Indeterminate) |
| Hb Hezhou | c.193G>A | 0 | 0.9728  (Pathogenic) | 26.5  (Supporting Pathogenic) | 8.68  (Supporting Pathogenic) | 0.999  (Moderate Pathogenic) | 0.67  (likely deleterious) | -0.03 | 0.948  (Strong Pathogenic) | 0  (Moderate Pathogenic) |
| Hb Burke | c.322G>C | 0.09 | 0.9951  (Strong Pathogenic) | 24.1  (Indeterminate) | - | - | 0.74  (likely deleterious) | - | 0.838  (Moderate Pathogenic) | 0  (Moderate Pathogenic) |
| Hb Crete | c.388G>C | 0.06 | 0.9586  (Moderate Pathogenic) | 21.2  (Supporting Benign) | - | - | 0.74  (likely deleterious) | - | 0.799  (Moderate Pathogenic) | 0.001  (Indeterminate) |
| β^CD81(CTC>CTA)^ | c.246C>A | 0 | - | 7.95  (Moderate Benign) | 0.959  (Supporting Benign) | - | - | -0.02 | - | - |
| β^IVSII-713(G>A)^ | c.315+713G>A | 0.08 | - | 0.028  (Strong Benign) | -0.235  (Moderate Benign) | - | - | - | - | - |

^a^Delta scores range from 0 to 1 and can be interpreted as the probability that the variant affects splicing at any position within a window around it (+/- 500bp by default). The SpliceAI score is provided for 0.2 (high recall), 0.5 (recommended), and 0.8 (high precision) cutoffs.

^b^AlphaMissense, CADD, PhyloP, PolyPhen, REVEL, and SIFT scores are based on thresholds and points established in Bergquist et al. 2024 and Pejaver et al. 2022.

^c^Gene-specific threshold is 0.46 on PrimateAI-3D.

^d^PromoterAI scores range from -1 to 1, with 0 meaning no activity. Negative values represent under-expression, and positive values represent over-expression. A threshold of ±0.1 is used for high sensitivity, and ±0.5 for high precision.

**Table S2** Numbers and proportions of β^0^-thalassemia, β^+^-thalassemia and unstable Hbs, normal β-globin gene, and errors for each Hb A_2_ level. Subjects with incomplete molecular analysis, Hb variants, and imprecise Hb A_2_ levels (range of data) were excluded.

| **%Hb A_2_** | **n** | **β^0^-thal**  **[n (%)]** | **β^+^-thal and unstable Hbs**  **[n (%)]** | **Normal**  **[n (%)]** | **Errors**  **[n (%)]** |
| --- | --- | --- | --- | --- | --- |
| 3.6 | 150 | 3 (2.0) | 19 (12.7) | 40 (26.7) | 88 (58.7) |
| 3.7 | 140 | 3 (2.1) | 19 (13.6) | 47 (33.6) | 71 (50.7) |
| 3.8 | 94 | 2 (2.1) | 25 (26.6) | 24 (25.5) | 43 (45.7) |
| 3.9 | 73 | 7 (9.6) | 24 (32.9) | 14 (19.2) | 28 (38.4) |
| 4.0 | 77 | 11 (14.3) | 25 (32.5) | 13 (16.9) | 28 (36.4) |
| 4.1 | 83 | 13 (15.7) | 42 (50.6) | 5 (6.0) | 23 (27.7) |
| 4.2 | 76 | 15 (19.7) | 37 (48.7) | 5 (6.6) | 19 (25.0) |
| 4.3 | 86 | 28 (32.6) | 47 (54.7) | 6 (7.0) | 5 (5.8) |
| 4.4 | 68 | 27 (39.7) | 31 (45.6) | 2 (2.9) | 8 (11.8) |
| 4.5 | 106 | 51 (48.1) | 43 (40.6) | 4 (3.8) | 8 (7.5) |
| 4.6 | 103 | 45 (43.7) | 48 (46.6) | 3 (2.9) | 7 (6.8) |
| 4.7 | 106 | 57 (53.8) | 39 (36.8) | 6 (5.7) | 4 (3.8) |
| 4.8 | 117 | 75 (64.1) | 38 (32.5) | 1 (0.9) | 3 (2.6) |
| 4.9 | 142 | 96 (67.6) | 36 (25.4) | 2 (1.4) | 8 (5.6) |
| 5.0 | 154 | 127 (82.5) | 23 (14.9) | 2 (1.3) | 2 (1.3) |
| 5.1 | 217 | 167 (77.0) | 44 (20.3) | 3 (1.4) | 3 (1.4) |
| 5.2 | 260 | 223 (85.8) | 33 (12.7) | 2 (0.8) | 2 (0.8) |
| 5.3 | 296 | 243 (82.1) | 50 (16.9) | 1 (0.3) | 2 (0.7) |
| 5.4 | 327 | 262 (80.1) | 63 (19.3) | 0 (0) | 2 (0.6) |
| 5.5 | 327 | 267 (81.7) | 57 (17.4) | 0 (0) | 3 (0.9) |
| 5.6 | 348 | 283 (81.3) | 63 (18.1) | 0 (0) | 2 (0.6) |
| 5.7 | 355 | 285 (80.3) | 66 (18.6) | 1 (0.3) | 3 (0.8) |
| 5.8 | 328 | 264 (80.5) | 61 (18.6) | 2 (0.6) | 1 (0.3) |
| 5.9 | 289 | 223 (77.2) | 65 (22.5) | 1 (0.3) | 0 (0) |
| 6.0 | 267 | 193 (72.3) | 70 (26.2) | 1 (0.4) | 3 (1.1) |
| 6.1 | 199 | 156 (78.4) | 42 (21.1) | 1 (0.5) | 0 (0) |
| 6.2 | 160 | 120 (75.0) | 36 (22.5) | 0 (0) | 4 (2.5) |
| 6.3 | 139 | 106 (76.3) | 32 (23.0) | 0 (0) | 1 (0.7) |
| 6.4 | 91 | 72 (79.1) | 18 (19.8) | 1 (1.1) | 0 (0) |
| 6.5 | 75 | 56 (74.7) | 19 (25.3) | 0 (0) | 0 (0) |
| 6.6 | 65 | 49 (75.4) | 15 (23.1) | 0 (0) | 1 (1.5) |
| 6.7 | 57 | 41 (71.9) | 10 (17.5) | 0 (0) | 6 (10.5) |
| 6.8 | 25 | 21 (84.0) | 2 (8.0) | 1 (4.0) | 1 (4.0) |
| 6.9 | 30 | 28 (93.3) | 1 (3.3) | 0 (0) | 1 (3.3) |
| 7.0 | 22 | 18 (81.8) | 3 (13.6) | 0 (0) | 1 (4.5) |
| ≥7.1 | 145 | 123 (84.8) | 12 (8.3) | 1 (0.7) | 9 (6.2) |
| **Total** | **5,597** | **3,760 (67.2)** | **1,258 (22.5)** | **189 (3.4)** | **390 (7.0)** |

**Figure S1** A couple with a risk of having homozygous β^0^-thalassemia fetus and confirmation of the Taiwanese β^0^-thalassemia by MLPA, GAP-PCR, and DNA sequencing. (**A**): Pedigree analysis of a couple with heterozygous Taiwanese β^0^-thalassemia. (**B**): MLPA analysis indicating a heterozygous deletion extending from probe HBB-1-148nt to HBB-Intr2-196nt with the threshold ratio at < 0.7. (**C**): GAP-PCR analysis using primers G9 and H5 for identification of the Taiwanese β^0^-thalassemia deletion (1,219 bp instead of a normal 2,576 bp). M represents the Lambda DNA/*Hind*III Marker. Lanes 1 and 3: normal control, and lanes 2 and 4: heterozygous Taiwanese β^0^-thalassemia. (**D**): DNA sequencing across the deletion breakpoint of the Taiwanese β^0^-thalassemia with NG_000007.3:g.69997_71353del.
