# Supplementary material for: Elevated Hemoglobin A2 : A Molecular Revisited, and Implications to β‐Thalassemia Screening
Source: J Clin Lab Anal. 2026 Jun 25:e70294. Online ahead of print. doi: 10.1002/jcla.70294 (PMC13399722; doi:10.1002/jcla.70294)
Supplement: Supplementary file 1 — Table S1: The in silico functional predictors of the five unstable Hbs and two novel β‐globin variants. Table S2: Numbers and proportions of β0‐thalassemia, β+‐thalassemia, and unstable Hbs, normal β‐globin gene, and errors for each Hb A2 level. Subjects with incomplete molecular analysis, Hb variants, and imprecise Hb A2 levels (range of data) were excluded. Figure S1: A couple with a risk of having a homozygous β0‐thalassemia fetus and confirmation of the Taiwanese β0‐thalassemia by MLPA, GAP‐PCR, and DNA sequencing. (A): Pedigree analysis of a couple with heterozygous Taiwanese β0‐thalassemia. (B): MLPA analysis indicating a heterozygous deletion extending from probe HBB‐1‐148 nt to HBB‐Intr2‐196 nt with the threshold ratio at < 0.7. (C): GAP‐PCR analysis using primers G9 and H5 for identification of the Taiwanese β0‐thalassemia deletion (1219 bp instead of a normal 2,576 bp). M represents the Lambda DNA/HindIII Marker. Lanes 1 and 3: Normal control, and lanes 2 and 4: Heterozygous Taiwanese β0‐thalassemia. (D): DNA sequencing across the deletion breakpoint of the Taiwanese β0‐thalassemia with NG_000007.3:g.69997_71353del. [file JCLA-9999-e70294-s001.docx]

**Table S1** The *in silico* functional predictors of the five unstable Hbs and two novel β-globin variants

| **Variants** | **HGVS name**  **(HBB)** | **SpliceAI^a^** | **AlphaMissense^b^** | **CADD^b^** | **PhyloP^b^** | **PolyPhen^b^** | **PrimateAI-3D^c^** | **PromoterAI^d^** | **REVEL^b^** | **SIFT^b^** |
| --- | --- | --- | --- | --- | --- | --- | --- | --- | --- | --- |
| Hb Dhonburi | c.380T>G | 0.04 | 0.2818  (Indeterminate) | 13.8  (Moderate Benign) | -2.04  (Moderate Benign) | 0.226  (Indeterminate) | 0.47  (likely deleterious) | - | 0.67  (Supporting Pathogenic) | 0.01  (Indeterminate) |
| Hb La Desirade | c.389C>T | 0.01 | 0.3754  (Indeterminate) | 22.6  (Supporting Benign) | 1.74  (Supporting Benign) | 0.025  (Supporting Benign) | 0.32  (Benign) | - | 0.818  (Moderate Pathogenic) | 0.04  (Indeterminate) |
| Hb Hezhou | c.193G>A | 0 | 0.9728  (Pathogenic) | 26.5  (Supporting Pathogenic) | 8.68  (Supporting Pathogenic) | 0.999  (Moderate Pathogenic) | 0.67  (likely deleterious) | -0.03 | 0.948  (Strong Pathogenic) | 0  (Moderate Pathogenic) |
| Hb Burke | c.322G>C | 0.09 | 0.9951  (Strong Pathogenic) | 24.1  (Indeterminate) | - | - | 0.74  (likely deleterious) | - | 0.838  (Moderate Pathogenic) | 0  (Moderate Pathogenic) |
| Hb Crete | c.388G>C | 0.06 | 0.9586  (Moderate Pathogenic) | 21.2  (Supporting Benign) | - | - | 0.74  (likely deleterious) | - | 0.799  (Moderate Pathogenic) | 0.001  (Indeterminate) |
| β^CD81(CTC>CTA)^ | c.246C>A | 0 | - | 7.95  (Moderate Benign) | 0.959  (Supporting Benign) | - | - | -0.02 | - | - |
| β^IVSII-713(G>A)^ | c.315+713G>A | 0.08 | - | 0.028  (Strong Benign) | -0.235  (Moderate Benign) | - | - | - | - | - |

^a^Delta scores range from 0 to 1 and can be interpreted as the probability that the variant affects splicing at any position within a window around it (+/- 500bp by default). The SpliceAI score is provided for 0.2 (high recall), 0.5 (recommended), and 0.8 (high precision) cutoffs.

^b^AlphaMissense, CADD, PhyloP, PolyPhen, REVEL, and SIFT scores are based on thresholds and points established in Bergquist et al. 2024 and Pejaver et al. 2022.

^c^Gene-specific threshold is 0.46 on PrimateAI-3D.

^d^PromoterAI scores range from -1 to 1, with 0 meaning no activity. Negative values represent under-expression, and positive values represent over-expression. A threshold of ±0.1 is used for high sensitivity, and ±0.5 for high precision.

**Table S2** Numbers and proportions of β^0^-thalassemia, β^+^-thalassemia and unstable Hbs, normal β-globin gene, and errors for each Hb A_2_ level. Subjects with incomplete molecular analysis, Hb variants, and imprecise Hb A_2_ levels (range of data) were excluded.

| **%Hb A_2_** | **n** | **β^0^-thal**  **[n (%)]** | **β^+^-thal and unstable Hbs**  **[n (%)]** | **Normal**  **[n (%)]** | **Errors**  **[n (%)]** |
| --- | --- | --- | --- | --- | --- |
| 3.6 | 150 | 3 (2.0) | 19 (12.7) | 40 (26.7) | 88 (58.7) |
| 3.7 | 140 | 3 (2.1) | 19 (13.6) | 47 (33.6) | 71 (50.7) |
| 3.8 | 94 | 2 (2.1) | 25 (26.6) | 24 (25.5) | 43 (45.7) |
| 3.9 | 73 | 7 (9.6) | 24 (32.9) | 14 (19.2) | 28 (38.4) |
| 4.0 | 77 | 11 (14.3) | 25 (32.5) | 13 (16.9) | 28 (36.4) |
| 4.1 | 83 | 13 (15.7) | 42 (50.6) | 5 (6.0) | 23 (27.7) |
| 4.2 | 76 | 15 (19.7) | 37 (48.7) | 5 (6.6) | 19 (25.0) |
| 4.3 | 86 | 28 (32.6) | 47 (54.7) | 6 (7.0) | 5 (5.8) |
| 4.4 | 68 | 27 (39.7) | 31 (45.6) | 2 (2.9) | 8 (11.8) |
| 4.5 | 106 | 51 (48.1) | 43 (40.6) | 4 (3.8) | 8 (7.5) |
| 4.6 | 103 | 45 (43.7) | 48 (46.6) | 3 (2.9) | 7 (6.8) |
| 4.7 | 106 | 57 (53.8) | 39 (36.8) | 6 (5.7) | 4 (3.8) |
| 4.8 | 117 | 75 (64.1) | 38 (32.5) | 1 (0.9) | 3 (2.6) |
| 4.9 | 142 | 96 (67.6) | 36 (25.4) | 2 (1.4) | 8 (5.6) |
| 5.0 | 154 | 127 (82.5) | 23 (14.9) | 2 (1.3) | 2 (1.3) |
| 5.1 | 217 | 167 (77.0) | 44 (20.3) | 3 (1.4) | 3 (1.4) |
| 5.2 | 260 | 223 (85.8) | 33 (12.7) | 2 (0.8) | 2 (0.8) |
| 5.3 | 296 | 243 (82.1) | 50 (16.9) | 1 (0.3) | 2 (0.7) |
| 5.4 | 327 | 262 (80.1) | 63 (19.3) | 0 (0) | 2 (0.6) |
| 5.5 | 327 | 267 (81.7) | 57 (17.4) | 0 (0) | 3 (0.9) |
| 5.6 | 348 | 283 (81.3) | 63 (18.1) | 0 (0) | 2 (0.6) |
| 5.7 | 355 | 285 (80.3) | 66 (18.6) | 1 (0.3) | 3 (0.8) |
| 5.8 | 328 | 264 (80.5) | 61 (18.6) | 2 (0.6) | 1 (0.3) |
| 5.9 | 289 | 223 (77.2) | 65 (22.5) | 1 (0.3) | 0 (0) |
| 6.0 | 267 | 193 (72.3) | 70 (26.2) | 1 (0.4) | 3 (1.1) |
| 6.1 | 199 | 156 (78.4) | 42 (21.1) | 1 (0.5) | 0 (0) |
| 6.2 | 160 | 120 (75.0) | 36 (22.5) | 0 (0) | 4 (2.5) |
| 6.3 | 139 | 106 (76.3) | 32 (23.0) | 0 (0) | 1 (0.7) |
| 6.4 | 91 | 72 (79.1) | 18 (19.8) | 1 (1.1) | 0 (0) |
| 6.5 | 75 | 56 (74.7) | 19 (25.3) | 0 (0) | 0 (0) |
| 6.6 | 65 | 49 (75.4) | 15 (23.1) | 0 (0) | 1 (1.5) |
| 6.7 | 57 | 41 (71.9) | 10 (17.5) | 0 (0) | 6 (10.5) |
| 6.8 | 25 | 21 (84.0) | 2 (8.0) | 1 (4.0) | 1 (4.0) |
| 6.9 | 30 | 28 (93.3) | 1 (3.3) | 0 (0) | 1 (3.3) |
| 7.0 | 22 | 18 (81.8) | 3 (13.6) | 0 (0) | 1 (4.5) |
| ≥7.1 | 145 | 123 (84.8) | 12 (8.3) | 1 (0.7) | 9 (6.2) |
| **Total** | **5,597** | **3,760 (67.2)** | **1,258 (22.5)** | **189 (3.4)** | **390 (7.0)** |


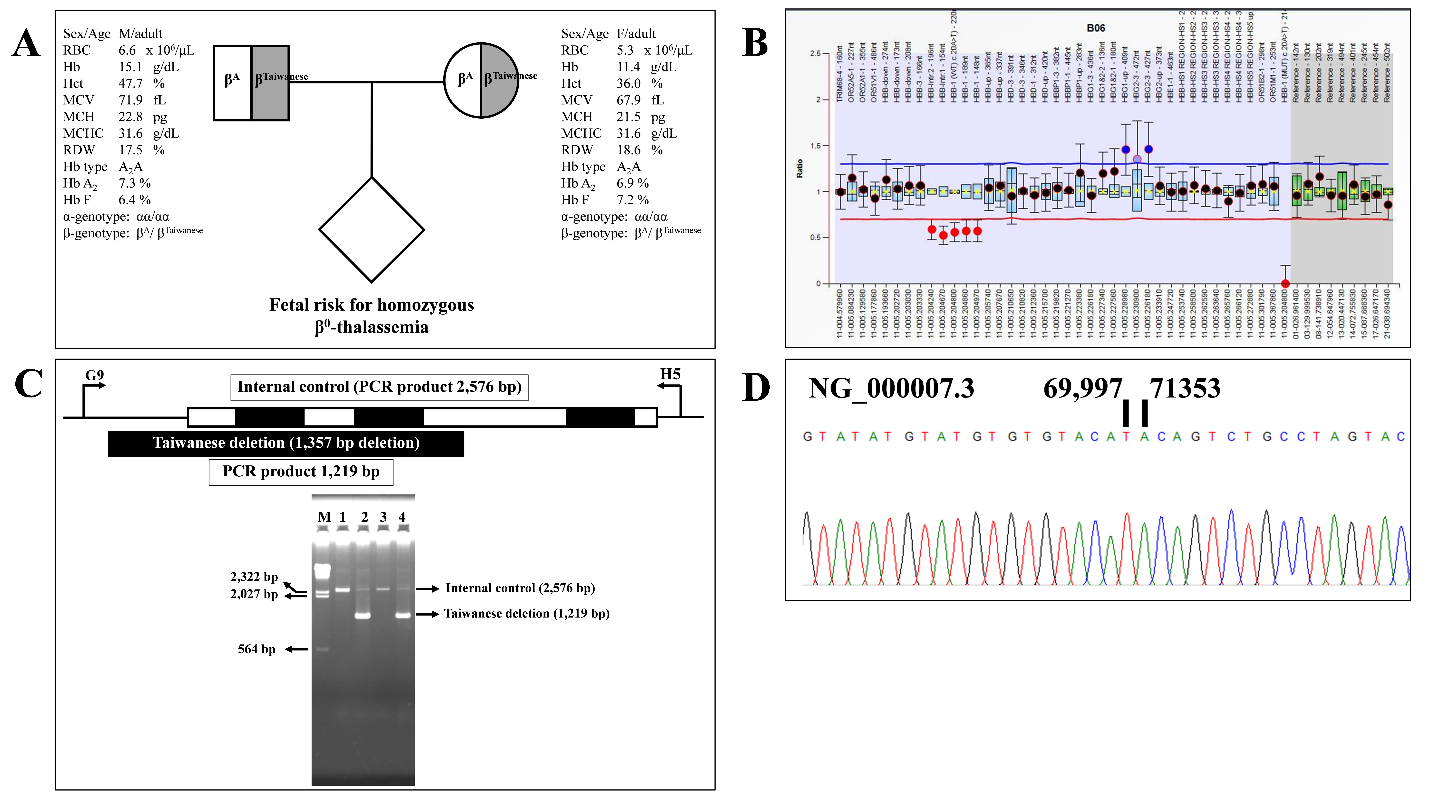


**Figure S1** A couple with a risk of having homozygous β^0^-thalassemia fetus and confirmation of the Taiwanese β^0^-thalassemia by MLPA, GAP-PCR, and DNA sequencing. (**A**): Pedigree analysis of a couple with heterozygous Taiwanese β^0^-thalassemia. (**B**): MLPA analysis indicating a heterozygous deletion extending from probe HBB-1-148nt to HBB-Intr2-196nt with the threshold ratio at < 0.7. (**C**): GAP-PCR analysis using primers G9 and H5 for identification of the Taiwanese β^0^-thalassemia deletion (1,219 bp instead of a normal 2,576 bp). M represents the Lambda DNA/*Hind*III Marker. Lanes 1 and 3: normal control, and lanes 2 and 4: heterozygous Taiwanese β^0^-thalassemia. (**D**): DNA sequencing across the deletion breakpoint of the Taiwanese β^0^-thalassemia with NG_000007.3:g.69997_71353del.
